# Designing a large field-of-view two-photon microscope using optical invariant analysis

**DOI:** 10.1117/1.NPh.5.2.025001

**Published:** 2018-02-19

**Authors:** Jonathan R. Bumstead, Jasmine J. Park, Isaac A. Rosen, Andrew W. Kraft, Patrick W. Wright, Matthew D. Reisman, Daniel C. Côté, Joseph P. Culver

**Affiliations:** a Washington University in Saint Louis, Department of Biomedical Engineering, St. Louis, Missouri, United States; b Washington University School of Medicine, Department of Radiology, St. Louis, Missouri, United States; c Washington University in Saint Louis, Department of Biology, St. Louis, Missouri, United States; d Washington University School of Medicine, Department of Neurology, St. Louis, Missouri, United States; e Washington University in Saint Louis, Department of Physics, St. Louis, Missouri, United States; f Université Laval, Génie Physique et Optique, Département de Physique, Ville de Québec, Quebec, Canada

**Keywords:** two-photon microscopy, scanning microscopy, optical design, optical invariant, etendue, neurophysiology

## Abstract

Conventional two-photon microscopy (TPM) is capable of imaging neural dynamics with subcellular resolution, but it is limited to a field-of-view (FOV) diameter <1  mm. Although there has been recent progress in extending the FOV in TPM, a principled design approach for developing large FOV TPM (LF-TPM) with off-the-shelf components has yet to be established. Therefore, we present a design strategy that depends on analyzing the optical invariant of commercially available objectives, relay lenses, mirror scanners, and emission collection systems in isolation. Components are then selected to maximize the space-bandwidth product of the integrated microscope. In comparison with other LF-TPM systems, our strategy simplifies the sequence of design decisions and is applicable to extending the FOV in any microscope with an optical relay. The microscope we constructed with this design approach can image <1.7-μm lateral and <28-μm axial resolution over a 7-mm diameter FOV, which is a 100-fold increase in FOV compared with conventional TPM. As a demonstration of the potential that LF-TPM has on understanding the microarchitecture of the mouse brain across interhemispheric regions, we performed *in vivo* imaging of both the cerebral vasculature and microglia cell bodies over the mouse cortex.

## 1 Introduction

Although two-photon microscopy (TPM) has revolutionized *in vivo* studies on the microarchitecture of the mouse cerebral cortex, it has primarily been limited to measuring brain dynamics over a field of view (FOV) of around $500\times 500\;{\mu m}^{2}$ (i.e., an FOV diameter of 707  μm).^1^^,^^2^ This limitation makes TPM impractical for the increasing number of studies on functional whole-brain imaging and mapping in mice that have been inspired by the effort to map the human connectome.^3^^,^^4^ Researchers have, therefore, primarily utilized mesoscopic optical imaging with planar illumination (MOIPI) techniques to study brain function over large regions of the mouse cortex (up to $10\times 10\;{\mathrm{mm}}^{2}$ FOV).^3^^,^^5^^,^^6^ However, MOIPI techniques have relatively poor resolution (around 200- to 300-μm lateral resolution) and depth penetration in comparison with TPM.^7^ As a result, the cellular dynamics of brain networks and large-scale neural phenomena in mice are still not well explored or understood. A potential way to help bridge the gap in studying the mouse brain across these spatial scales is to extend the FOV in TPM while maintaining cellular resolution.

Although there have been advancements in TPM design, many of the developments have focused on imaging faster^8^ and deeper into tissue^9^ using adaptive optics^10^ and manipulation of the point spread function (PSF) with temporal focusing,^11^ microlens arrays,^12^ and diffractive optical elements.^13^ Only recently have researchers turned attention to extending the FOV.^14^^–^^16^ The FOV in TPM is limited primarily by high magnification objective lenses, poorly designed relay lenses, and small field collection systems.^14^^,^^16^^,^^17^ The use of high magnification objectives stems from resolution and signal requirements that demand objective lenses with numerical aperture (NA) >0.7. As a result, this biases microscope designs to include objectives with focal lengths <9  mm (i.e., magnification greater than 20×), which make imaging field diameters >1  mm difficult to achieve.^18^^–^^21^

The less explored and more technically challenging problem in extending the FOV is the relay lenses. Interestingly, there has been greater emphasis on improving the performance of objective lenses, typically high-NA high-magnification objectives, than relay lenses used in laser scanning microscopy systems. Therefore, the current FOV in many commercially available and custom-built two-photon and confocal microscopes is actually limited due to the use of achromatic doublets as relay lenses, not the objective lens.^16^^,^^17^

Our goal for this study was to overcome these design challenges by applying a principled design approach that enabled us to evaluate the performance of individual components and compare them with the demands of the objective lens. The approach relies on analyzing the optical invariant, which has been generally underutilized in the design of TPM systems. Here, we present a LF-TPM system constructed with off-the-shelf components and only a single scanning relay, which reduces the cost (<40,000 USD beyond the initial expense of the TiSapphire laser) and complexity while still maintaining similar resolution and FOV to other more expensive LF-TPM systems previously reported.^14^^,^^15^ To demonstrate the capabilities of the system for multiscale imaging of the mouse brain, we imaged the cerebral vasculature and microglia cell bodies over a 7-mm-diameter region of the mouse cortex with <1.7-μm lateral and <28-μm axial resolution.

## 2 Design

In its simplest form, a two-photon microscope consists of a scanning system in the x- and y-directions that is conjugated to the rear of an objective lens. Conjugation of these planes is achieved with an afocal relay consisting of two lenses, often called the scan and tube lenses. Analysis of a ray diagram of this system is the first step in redesigning the microscope for large FOV imaging. However, as several components are integrated into the system, it can become difficult to make design decisions and isolate components using raytracing techniques. In contrast, analyzing the conservation of radiant power by calculating the optical invariant in the system can eliminate intermediate raytracing calculations, provide an intuitive understanding of component requirements, and enable several design decisions to be made before detailed system optimization or custom component design.^22^^,^^23^

The optical invariant is a constant conserved throughout an ideal aberration-free optical system, which is calculated with the height and angle of the chief and marginal rays. Although the optical invariant can be calculated at any transverse plane in an optical system, it is especially useful for comparing the angle and displacement of rays at aperture and field planes. Often this concept of an optical system’s light acceptance is described by the invariant’s three-dimensional (3-D) analog, the throughput, or étendue, which is proportional to the square of the invariant.^22^ Throughput and the optical invariant are conceptually interchangeable.

Throughout this report, we calculate the optical invariant of isolated optical components and integrated optical systems consisting of several components. In general, the optical invariant of an integrated system will be limited by the component with the lowest optical invariant in isolation. For example, if a galvanometer mirror has a lower invariant in isolation in comparison with an objective, then the optical invariant of a system consisting of both components will be equal to the invariant of the galvanometer. In this case, the objective would not be imaging to its full capabilities as specified by manufacturers. Therefore, our approach for designing an LF-TPM system was to first select a high-throughput objective lens and then identify optical components capable of supporting an invariant equal to or greater than the invariant of the objective in isolation.

The performance of many off-the-shelf components required for TPM is specified for a single operating condition. Therefore, full characterization of the component requires measuring the performance as a function of input beam diameter using optical engineering software. After evaluating isolated components and selecting candidates for LF-TPM, we tested integrated microscopy systems, first with optical engineering software (OpticStudio, Zemax, Kirkland) and then experimentally with fluorescent beads.

### 2.1 Optical Invariant Analysis of TPM Systems

**Fig. 1:**
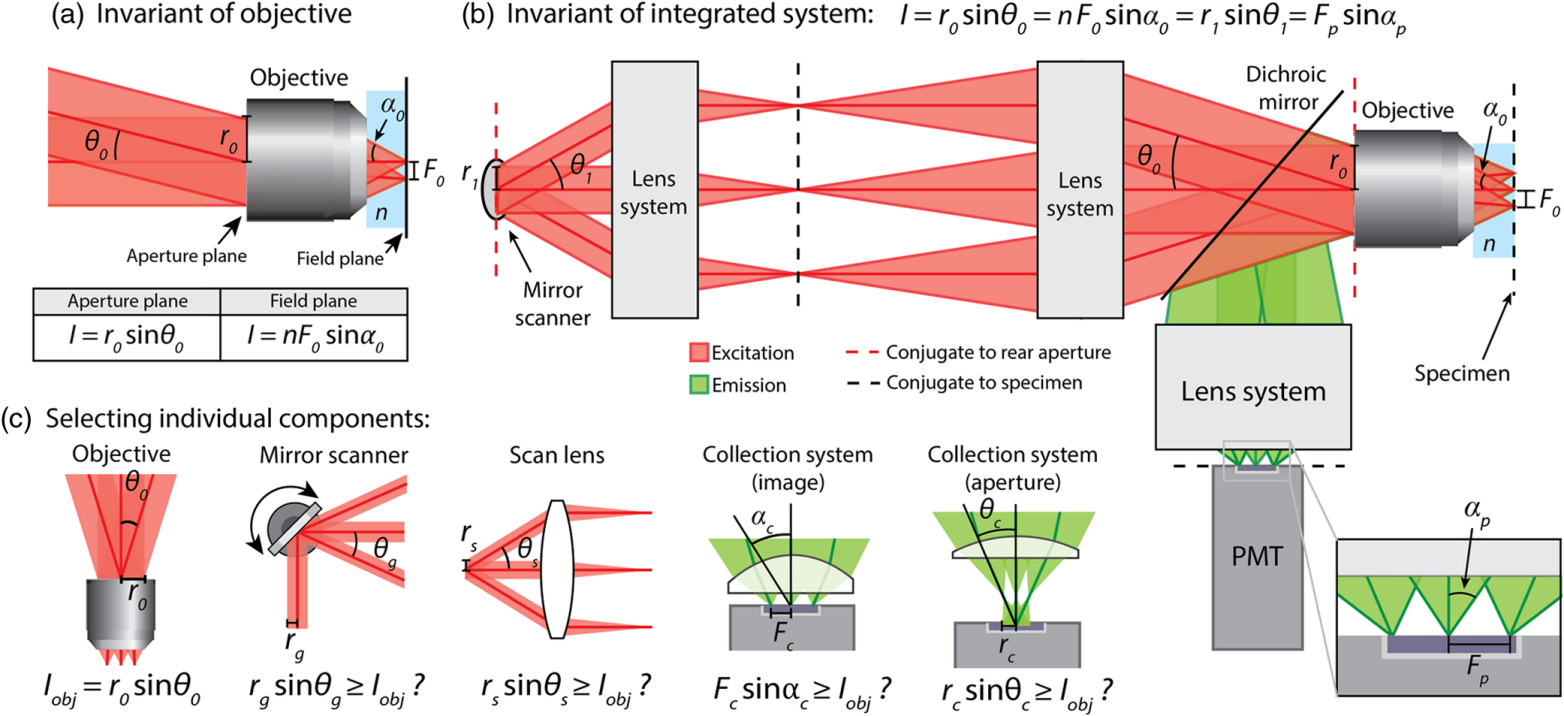
Optical invariant in laser scanning two-photon microscopy. (a) Optical invariant defined at aperture plane and field plane of isolated objective lens. A collimated beam with radius $r_{o}$ is directed to the rear of an objective that focuses the beam a distance $F_{0}$ from the optical axis at the specimen plane. $F_{0}$ depends on the angle of the collimated beam $\theta_{0}$. The optical invariant is equal at the aperture and field planes. (b) Basic laser scanning two photon microscope. A laser beam is scanned with a mirror scanner and relayed to the rear aperture of an objective with two lens systems. The optical invariant of the objective is equal to the invariant at the mirror scanner and the photocathode in an aberration free, lossless system. (c) Individual components in the microscope can be isolated by comparing the invariant of the objective to the invariant of the component. For the system to be limited by objective performance, invariant of isolated component must be greater than or equal to invariant of objective.

We analyzed the conservation of radiant power of a basic laser scanning two-photon microscope by first considering the optical invariant at the rear aperture and front focal plane of an isolated objective lens

$$I=r_{0}\;\sin\;\theta_{0}=nF_{0}\;\sin\;\alpha_{0},$$ (1)

where I is the optical invariant, $r_{0}$ and $\theta_{0}$ are the beam radius and incident angle of collimated light at the rear of the objective, respectively, $F_{0}$ and $\alpha_{0}$ are the FOV radius and angle of the cone of light at the image plane, respectively, and n is the index of refraction of the immersion fluid of the objective[Fig. 1(a)].^22^^–^^24^ These parameters are usually defined by manufacturers in terms of the objective’s numerical aperture (NA), magnification, and field number, which we relate to $F_{0}$, $\theta_{0}$, $\alpha_{0}$, and n in the next section. Equation (1) assumes that the optical system is aplanatic and is a more accurate definition of the optical invariant for microscopy systems in comparison with the paraxial definition (Appendix A).^24^^,^^25^ Depending on the manufacturer and objective lens, the plane located at the rear of the objective may be defined as the back focal plane, back aperture, or pupil plane. To avoid confusion with these terms, we define the plane at the rear of the objective near the threading as the “rear aperture.” For Olympus objectives, the rear aperture has a diameter equal to the pupil diameter as defined in Sec. 2.2. In many laser scanning microscopes, the scanners are conjugate to this position, which may not coincide with the back focal plane of the objective lens.^17^^,^^18^

Consider an ideal integrated laser scanning microscope with invariant equal to the invariant of the objective in isolation. To scan the FOV of the objective, a laser beam with radius $r_{1}$ incident upon a mirror scanner (e.g., galvanometer) is relayed onto the rear aperture of an objective lens [Fig. 1(b)]. The full resolution of the objective lens is achieved by expanding the beam to fill the rear aperture. Emission from the sample is then separated with a dichroic mirror and collected onto a photodetector, typically a photomultiplier tube (PMT).

At this point, it is useful to analyze the optical invariant at conjugate aperture planes, which are the rear aperture plane of the objective and the mirror scanner plane. Thus,

$$r_{0}\;\sin\;\theta_{0}=r_{1}\;\sin\;\theta_{1},$$ (2)

where $r_{0}$ and $\theta_{0}$ are the beam radius and angle of collimated light at the rear aperture, respectively, and $r_{1}$ and $\theta_{1}$ are the beam radius and scan angle at the mirror scanner, respectively. This simple relationship provides an intuitive guide for comparing the demands of the objective lens with that of other components (e.g., galvanometers and relay lenses) in the microscope.

The challenge of designing an emission collection system for LF-TPM can also be approached by comparing the optical invariant at the specimen and the PMT photocathode

$$nF_{0}\;\sin\;\alpha_{0}=F_{p}\;\sin\;\alpha_{p},$$ (3)

where $F_{0}$ and $\alpha_{0}$ are the FOV radius and angle of the cone of emission light entering the objective, respectively, and $F_{p}$ and $\alpha_{p}$ are the sensor radius and angle of the cone of light exiting the collection optics, respectively [Fig. 1(b)].^14^^,^^22^ Alternatively, the collection system can be designed by considering the photocathode of the PMT conjugate to the rear aperture of the objective by placing the photocathode at the exit pupil of the collection system.^26^ For the system to be limited by the objective, the component’s invariant in isolation must be greater than or equal to the invariant of the objective lens [Fig. 1(c)].

### 2.2 LF-TPM Requires Objectives Lenses with High Throughput

Before selecting relay lenses and a scanner for the microscope, we needed to identify commercially available objectives suitable for large FOV imaging, which requires expressing the optical invariant of objective lenses in terms of parameters provided by manufacturers. The FOV of commercially available infinity-corrected objective lenses is usually defined with reference to a tube lens that forms an image conjugate to the specimen plane. Objective manufacturers define the maximum diameter of this image, known as the field number, such that certain resolution and intensity requirements are fulfilled over the FOV (e.g., diffraction limited and no vignetting over FOV).^16^^,^^27^

The corresponding FOV that is imaged at the specimen depends on the magnification of the objective lens and tube lens system, which is defined for an infinity-corrected objective as

$$M=\frac{f_{t}}{f_{o}},$$ (4)

where M is the magnification of the objective lens, $f_{t}$ is the focal length of the tube lens for which the objective is designed, and $f_{o}$ is the focal length of the objective. Generally, $f_{t}$ is equal to 180 mm for Olympus objectives and 200 mm for Nikon objectives. The diameter of the FOV ($2F_{0}$) that an objective lens is designed to image is thus

$$2F_{0}=\frac{\mathrm{FN}}{M},$$ (5)

where $F_{0}$ is the FOV radius and FN is the field number. The angle of collimated light at the rear aperture required for focusing light at a position $F_{0}$ from the optical axis can also be defined in terms of objective parameters

$$\theta_{0}=\sin^{-1}(\frac{F_{0}}{f_{o}})=\sin^{-1}(\frac{\mathrm{FN}}{2f_{t}}),$$ (6)

where $\theta_{0}$ is the angle of collimated light at the rear aperture. By analyzing the optical invariant of the objective lens in isolation [see Fig. 1(a) and Appendix A], it is also apparent that the beam radius at the rear aperture $r_{0}$ required to achieve the full NA of the objective is

$$d_{\mathrm{pupil}}=2r_{0}=\frac{2f_{t}\mathrm{NA}}{M},$$ (7)

where $d_{\text{pupil}}$ is known as the pupil diameter of the objective and NA is the numerical aperture, which is defined as $\mathrm{NA}=n\;\sin\;\alpha_{0}$.

Finally, the optical invariant of an objective lens can be expressed in terms of parameters provided by manufacturers by either plugging Eq. (5) into Eq. (1) or plugging Eqs. (6) and (7) into Eq. (1)

$$I=\frac{\mathrm{NA}\cdot \mathrm{FN}}{2M},$$ (8)

where I is the optical invariant of the isolated objective.

**Fig. 2:**
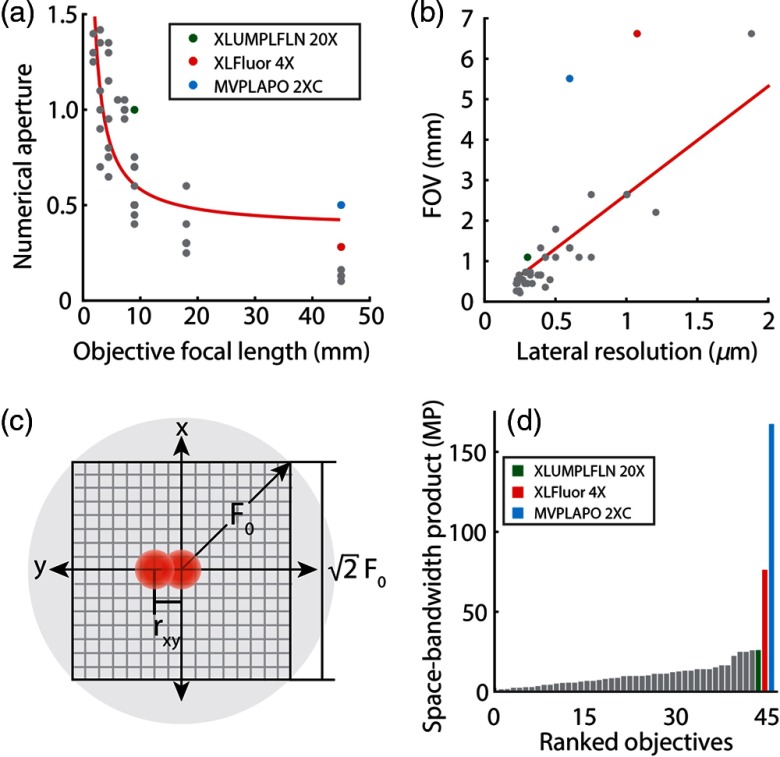
Selecting objective lenses for LF-TPM. (a) Numerical aperture plotted against objective focal length for 45 commercially available Olympus objectives. Red curve is the best fit for the NA’s inverse relation to the focal length of the objective. (b) The FOV diameter plotted as a function of the resolution of the objective lens. Two objectives are candidates for LF-TPM (XLFLUOR4X and MVPLAPO 2XC). Red curve is the best fit for the FOV’s linear relation to the lateral resolution. (c) Diagram of circular FOV (gray) and intensity PSF squared (pink). The rectangular FOV and pixels required for sufficient sampling are also shown. (d) The space-bandwidth product of the 45 objective lenses.

To find candidate objectives for LF-TPM, we compared the specifications of 45 commercially available Olympus objectives (Olympus, Tokyo, Japan) (Appendix B and Fig. 2). For this analysis, the lateral resolution of a two-photon microscope was defined as the full width at half maximum (FWHM) of the excitation PSF squared

$$r_{xy}=\{\begin{array}{ll} 0.320\sqrt{2\ln2}\frac{\lambda_{\mathrm{ex}}}{\mathrm{NA}} & \mathrm{NA}\leq 0.7\\ 0.325\sqrt{2\ln2}\frac{\lambda_{\mathrm{ex}}}{{\mathrm{NA}}^{0.91}} & \mathrm{NA}>0.7 \end{array},$$ (9)

where NA is the numerical aperture of the objective and $\lambda_{ex}$ is the excitation wavelength, which was set to 800 nm.^20^ As expected, the NA generally follows an inverse relationship with the focal length of the objective with deviations due to differences in the pupil diameter of the objectives [Fig. 2(a)]. By plotting the resolution versus FOV using Eqs. (5) and (9), we were able to find objectives that deviated from the approximately linear trade-off between FOV and resolution [Fig. 2(b)]. The Olympus XLFLUOR4X (NA 0.28, f=45  mm) and MVPLAPO 2XC (NA 0.5, f=45  mm) macro-objectives provide a large FOV (>5  mm) while maintaining subcellular resolution in comparison with all objectives analyzed and were therefore selected for developing LF-TPM. For reference, an objective lens commonly used for *in vivo* TPM, the Olympus XLUMPLFLN 20X (NA 1.0, f=9  mm), is also labeled.

To assess the amount of information that could be transmitted by these objective lenses, the space-bandwidth product (SBP) was also calculated using

$$\mathrm{SBP}=\frac{{\left(\sqrt{2}F_{0}\right)}^{2}}{{\left(r_{xy}/2\right)}^{2}}=8{\left(\frac{F_{0}}{r_{xy}}\right)}^{2},$$ (10)

where SBP is the space-bandwidth product, $F_{0}$ is the FOV radius, and $r_{xy}$ is the lateral resolution. The SBP can be conceptualized as the maximum number of pixels (i.e., information) that can be transmitted by an objective lens or imaging system [Fig. 2(c)] and is calculated as the area of the FOV divided by the area of a pixel. To prevent aliasing, the image plane should be sampled such that the pixel length is less than or equal to half the FWHM of the lateral PSF. In Eq. (10), the SBP is calculated using the rectangular FOV so that it is more easily comparable with existing microscopy systems. One side of the rectangular FOV can be obtained by multiplying the FOV radius, $F_{0}$, by √2. The macro-objectives considered for LF-TPM can potentially transmit 3 to 6 times more information than other commercially available objectives that we analyzed [Fig. 2(d)].

By plugging Eq. (5) into Eq. (10) and approximating the lateral resolution as $r_{xy}\approx 0.38\frac{\lambda_{ex}}{\mathrm{NA}}$ for all NA, we find that the SBP is proportional to the optical invariant squared (i.e., the throughput)

$$\mathrm{SBP}\approx 55.4{\left(\frac{\mathrm{FN}\cdot \mathrm{NA}}{2M\lambda_{\mathrm{ex}}}\right)}^{2}=55.4{\left(\frac{I}{\lambda_{\mathrm{ex}}}\right)}^{2}.$$ (11)

In other words, higher throughput objectives have the potential to transmit more information than lower throughput objectives. Optimization of the optical invariant, therefore, serves as a tool to increase the SBP of the microscope, which is critical for developing multiscale imaging tools.

### 2.3 Applying the Optical Invariant for Determining Galvanometers Suitable for LF-TPM

With the two candidate objective lenses selected, the optical invariant of potential scanners was evaluated to determine which components could support the objectives. The Olympus XLFLUOR4X (NA 0.28, M=4, FN=26.5) has a pupil diameter of 25.2 mm, max field angle of 4.2 deg, and optical invariant of 0.92 mm. The MVPLAPO 2XC (NA 0.5, M=4, FN=22) has a pupil diameter of 45 mm, max field angle of 3.5 deg, and optical invariant of 1.37 mm. For a system to not be limited by the scanner, the optical invariant of the scanner in isolation must be greater than or equal to the optical invariant of these objectives.

**Fig. 3:**
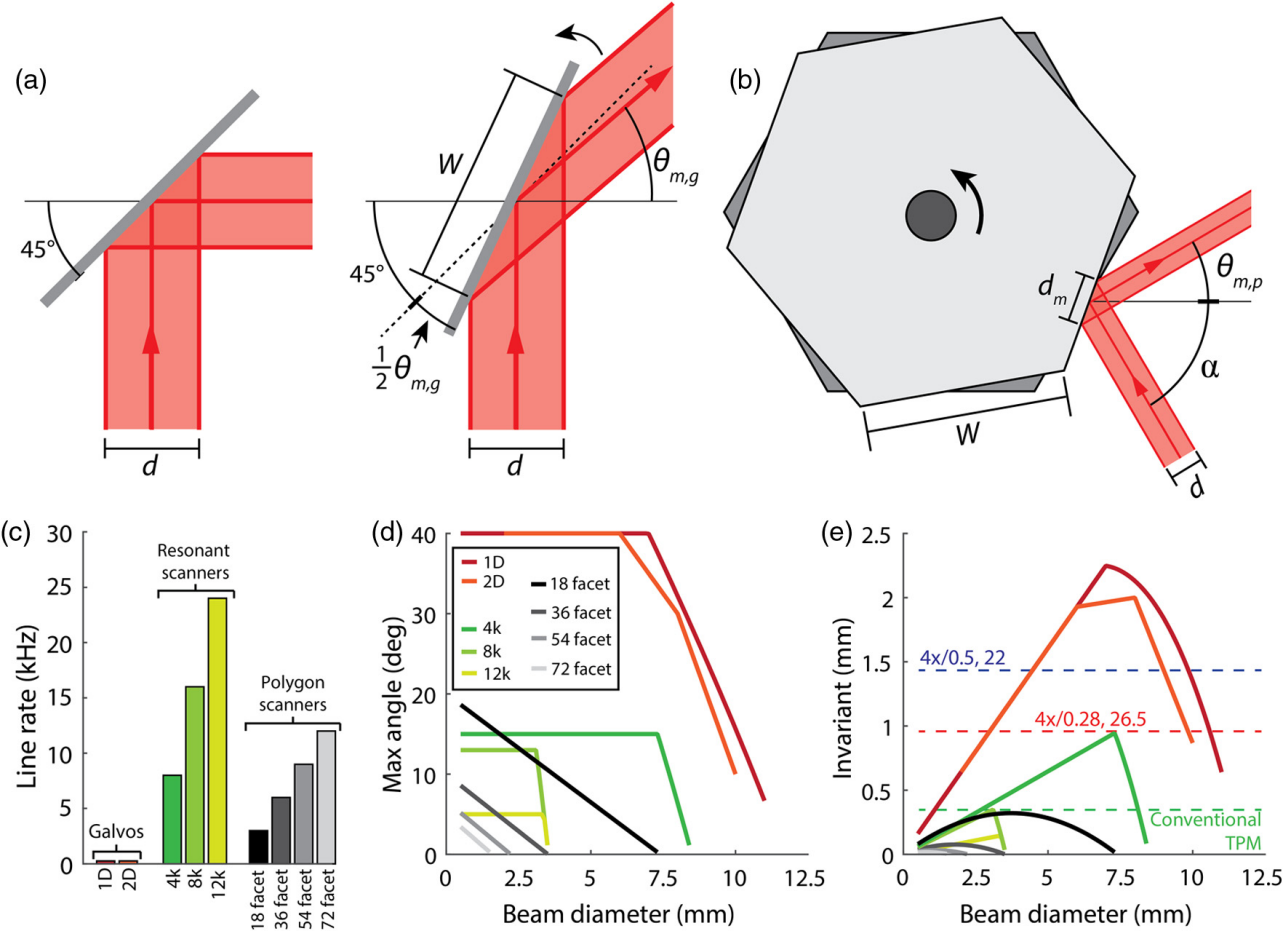
Evaluating performance of mirror scanners. (a) Diagram of galvanometer or resonant scanner with input beam diameter d. Given d and the clear aperture of the galvanometer W, the maximum scan angle $\theta_{m,g}$ can be determined. (b) Diagram of spinning polygonal scanner with clear aperture W, input beam diameter d, beam footprint on the facet $d_{m}$, and feed angle α. (c) Line rate of galvanometer mirrors, resonant scanners, and polygonal scanners. Parts are listed in Appendix B. (d) Maximum scan angle of scanners plotted as a function of input beam diameter for the nine scanners calculated using Eqs. (12) and (15). (e) Optical invariant of scanners in isolation plotted as a function of input beam diameter to scanner. Curves are colored according to legend in (d). Also shown is the optical invariant of a conventional TPM system (dashed green line) and the two macro-objectives: XLFLUOR4X (dashed red line) and MVPLAPO 2XC (dashed blue line). Comparison of (c) and (e) demonstrates trade-off between speed and throughput.

We analyzed the optical invariant of traditional galvanometer mirrors, resonant scanners, and polygonal scanners by calculating the maximum scan angle as a function of input beam diameter to the scanner. Consider a beam with diameter d incident upon a galvanometer or resonant scanner at a 45-deg angle [Fig. 3(a)]. As the scan angle increases, the beam footprint on the scanner also increases. Vignetting of the beam occurs when the beam footprint exceeds the clear aperture of the scanner. Therefore, the maximum scan angle will be limited by either the scanner’s maximum rotation angle or vignetting

$$\theta_{m,g}(d)=\{\begin{array}{ll} 2[\cos^{-1}(\frac{d}{W})-45] & \theta_{m,g}<\theta_{\max}\\ \theta_{\max} & \theta_{m,g}\geq \theta_{\max} \end{array},$$ (12)

where $\theta_{m,g}$ is the maximum optical scan angle of a galvanometer or resonant scanner with respect to the optical axis, d is the input beam diameter, W is the clear aperture, and $\theta_{\max}$ is the maximum allowable optical scan angle.

Similarly, the maximum scan angle of polygonal scanners can be calculated as a function of input beam diameter to the scanner [Fig. 3(b)]. The beam footprint on the scanner depends on the feed angle

$$d_{m}=\frac{1.5d}{\cos(\alpha /2)},$$ (13)

where $d_{m}$ is the diameter of the beam footprint on the scanner, d is the input beam diameter, and α is the feed angle. The factor of 1.5 is required for maintaining nearly uniform intensity across the scan. For polygonal scanners, the duty cycle of the scan, which is the ratio of active scan time to the total time that the beam is incident on a facet, must be calculated for determining the maximum scan angle

$$C=1-\frac{d_{m}}{W},$$ (14)

where C is the duty cycle and W is the width of facet. Finally, the maximum scan angle can be expressed as a function of input beam diameter to the polygonal scanner

$$\theta_{m,p}(d)=\frac{360C}{N}=\frac{360}{N}[1-\frac{1.5d}{W\cos(\alpha /2)}],$$ (15)

where $\theta_{m,p}$ is the maximum optical scan angle of a polygonal scanner, C is the duty cycle, N is the number of facets, and d is the input beam diameter to the polygon.^28^

A total of nine commercially available scanners were analyzed (Appendix B) by first comparing the line rate of the scanners [Fig. 3(c)]. Using Eqs. (12) and (15), we then plotted the maximum scan angle as a function of input beam diameter to the scanners [Fig. 3(d)]. For the two-dimensional (2-D) galvanometer, we used data provided by the manufacturer. To determine whether or not the scanners would limit the performance of the integrated microscope, we also compared the optical invariant of the isolated scanners to the macro-objective lenses [Fig. 3(e)]. The optical invariant was plotted as a function of input beam diameter with the data from Fig. 3(d). In addition to comparing the invariant of mirror scanners to macro-objectives, we also included the invariant of a conventional TPM system by calculating the invariant of a microscope capable of diffraction-limited imaging over an FOV diameter of 707  μm with the Olympus XLUMPLFLN 20× [i.e., I=9 sin(2.25  deg)=0.35  mm]. While resonant and polygonal mirror scanners are faster than traditional galvanometer mirrors, they generally have lower throughput that does not match the demands of the objectives selected for LF-TPM. For this large FOV microscope, we, therefore, chose to use traditional galvanometer mirrors, not resonant or polygonal scanners.

### 2.4 Calculating the Optical Invariant of Isolated Relay Lenses

**Fig. 4:**
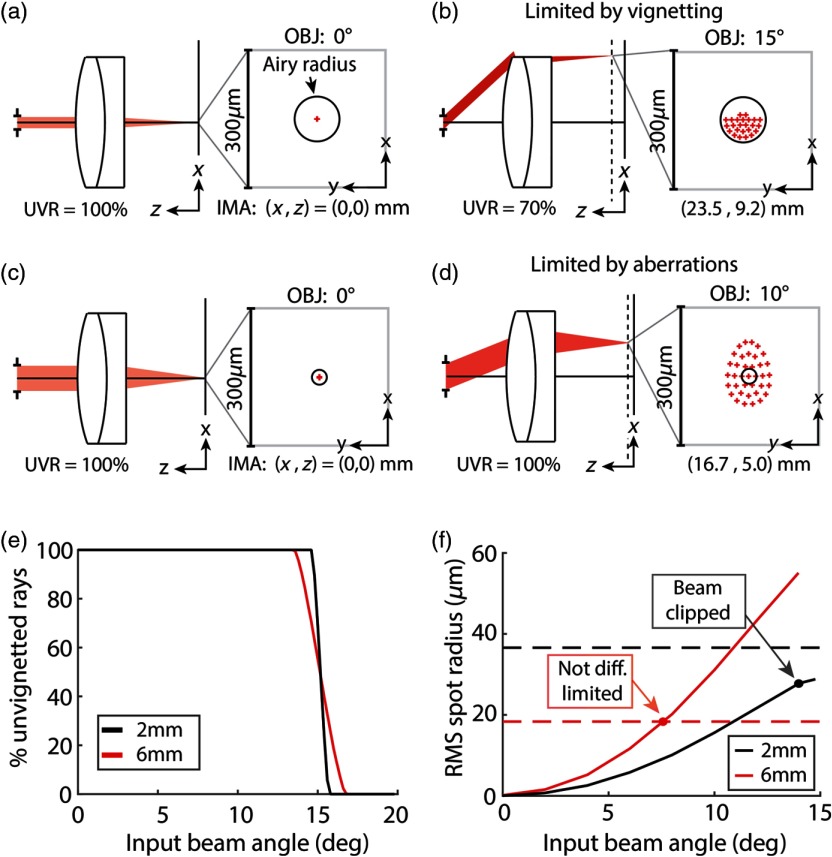
Optical aberrations or vignetting limit the optical throughput of relay lenses. (a) Layout and spot diagram of achromatic doublet (AC508-100-B, Thorlabs) modeled in Zemax. The 2-mm-diameter beam is focused on axis. The spot size is diffraction-limited, as indicated by the spot size predicted by ray optics being less than the Airy radius. There are 100% unvignetted rays (UVR) at the image plane. Layout is not to scale. (b) Same as (a), but for scan angle of 15 deg. 30% of the rays are clipped and do not reach the image plane. (c) Layout and spot diagram for the same achromatic doublet with input beam diameter of 6 mm. (d) Same as (c), but for scan angle of 10 deg. The beam is not diffraction-limited at the image plane. (e) Percent of unvignettted rays as a function of input beam angle for 2-mm- and 6-mm-input beam diameter. Data show maximum scan angle possible before vignetting. (f) RMS spot size radius at the image plane as a function of input beam angle. Dashed black and red lines are diffraction limit for 2-mm- and 6-mm-input beam diameter, respectively. The performance of the achromatic doublet is limited by vignetting for input beam diameter equal to 2 mm, whereas it is limited by optical aberrations when the input beam diameter is equal to 6 mm.

Similar to our selection of beam scanners for LF-TPM, we needed to identify relay lenses that could support the invariant of the isolated macro-objective lenses. The max scan angle of a relay lens is typically limited by vignetting at small input beam diameters [Figs. 4(a) and 4(b)] and optical aberrations at large input beam diameters [Figs. 4(c) and 4(d)]. We, therefore, defined the diffraction-limited max scan angle for a given input beam diameter as the beam angle that could be scanned before the beam was either clipped or was no longer diffraction-limited at the field plane. A full pipeline of our analysis for determining the optical invariant as a function of input beam diameter for isolated relay lenses is included in Appendix B of this report.

As an example, we analyzed a NIR achromatic doublet [effective focal length (efl)=100  mm; AC508-100-B, Thorlabs, Newton] in telecentric configuration using Zemax. The percentage of unvignetted rays was plotted as a function of beam angle for input beam diameters of 2 and 6 mm to determine at which scan angle the beam was clipped by the lens [Fig. 4(e)]. To analyze the performance at the field plane, the RMS spot radius, which is representative of the resolution predicted by ray optics, was plotted as a function of scan angle for the two input beam diameters [Fig. 4(f)]. If the RMS spot radius is greater than the Airy radius, then the resolution is limited by optical aberrations.^22^ When the input beam diameter is 2 mm, the RMS spot radius is less than the Airy radius and the max scan angle is limited by vignetting. Conversely, the max scan angle achievable for an input beam diameter of 6 mm is limited due to optical aberrations (i.e., the RMS spot radius exceeds the Airy radius before the beam is clipped).

**Fig. 5:**
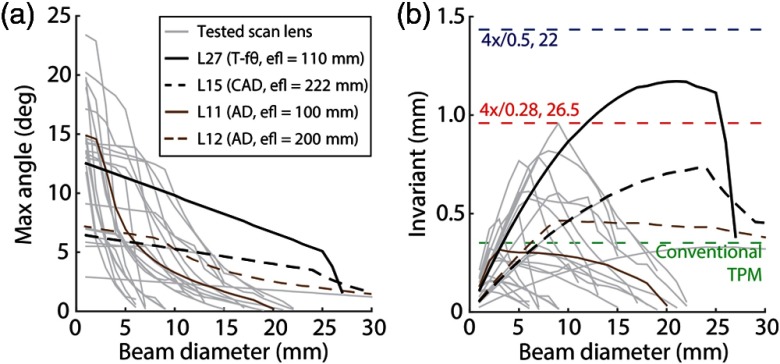
Performance of commercially available relay lenses. (a) Max scan angle as a function of input beam diameter for 27 relay lenses modeled in Zemax. Max scan angle is limited by either vignetting or optical aberrations. Highlighted are the best performing telecentric f-theta (T-fθ) scan lens (L27), best performing compound achromatic doublet (CAD) design (L15), and two achromatic doublet (AD) lenses that are typically used in conventional TPM (efl=100  mm; L11 and efl=200  mm; L12). All relay lenses and corresponding labels are listed in Appendix B. (b) Optical invariant of the 27 relay lenses plotted as a function of input beam diameter. Curves are colored according to legend in (a). The optical invariant for a conventional TPM system (dashed green) and the two macro-objectives shown in Fig. 2 are also labeled (dashed red and dashed blue lines). These results show at which beam diameter a relay lens has the best performance. Candidate relay lenses for LF-TPM should have optical invariant that is comparable with the macro-objective lenses.

The example in Fig. 4 highlighted the performance of one achromatic doublet at two operating conditions (i.e., input beam diameters of 2 and 6 mm). We extended the analysis to determine the max scan angle and optical invariant over a range of input beam diameters for 26 additional relay lenses with focal lengths ranging from 18 to 500 mm (Fig. 5). The analysis was conducted using Zemax and included a variety of designs including plano-convex lenses, NIR achromatic doublets, compound achromatic doublets, and telecentric f-theta scan lenses (Appendix B). The calculations yield curves that relate the diffraction-limited max scan angle to input beam diameter [Fig. 5(a)]. To better compare the performances of the relay lenses to the objective lens and make design decisions, we also determined the isolated relay lenses’ invariant as a function of input beam diameter [Fig. 5(b)].

Of the 27 lenses, we highlighted two of the best performing: a compound achromatic doublet lens system (efl=222  mm; AC508-400-B and AC508-500-B, Thorlabs) and a telecentric f-theta scan lens (efl=115  mm; 64-422, Edmund Optics, Barrington) [Fig. 5(b)]. These lenses are given the labels of L15 and L27, respectively (Appendix B). Both L15 and L27 are close to matching the invariant of the macro-objective lenses. In comparison, achromatic doublets (efl=100  mm; AC508-100-B and efl=200  mm; AC508-200-B, Thorlabs) match the invariant required for conventional TPM but are unable to support the demands of the macro-objective lenses and therefore generally fail as relay lenses in LF-TPM. These achromatic doublets with focal lengths of 100 and 200 mm are abbreviated as L11 and L12, respectively.

### 2.5 Testing Optical Relays for LF-TPM

**Fig. 6:**
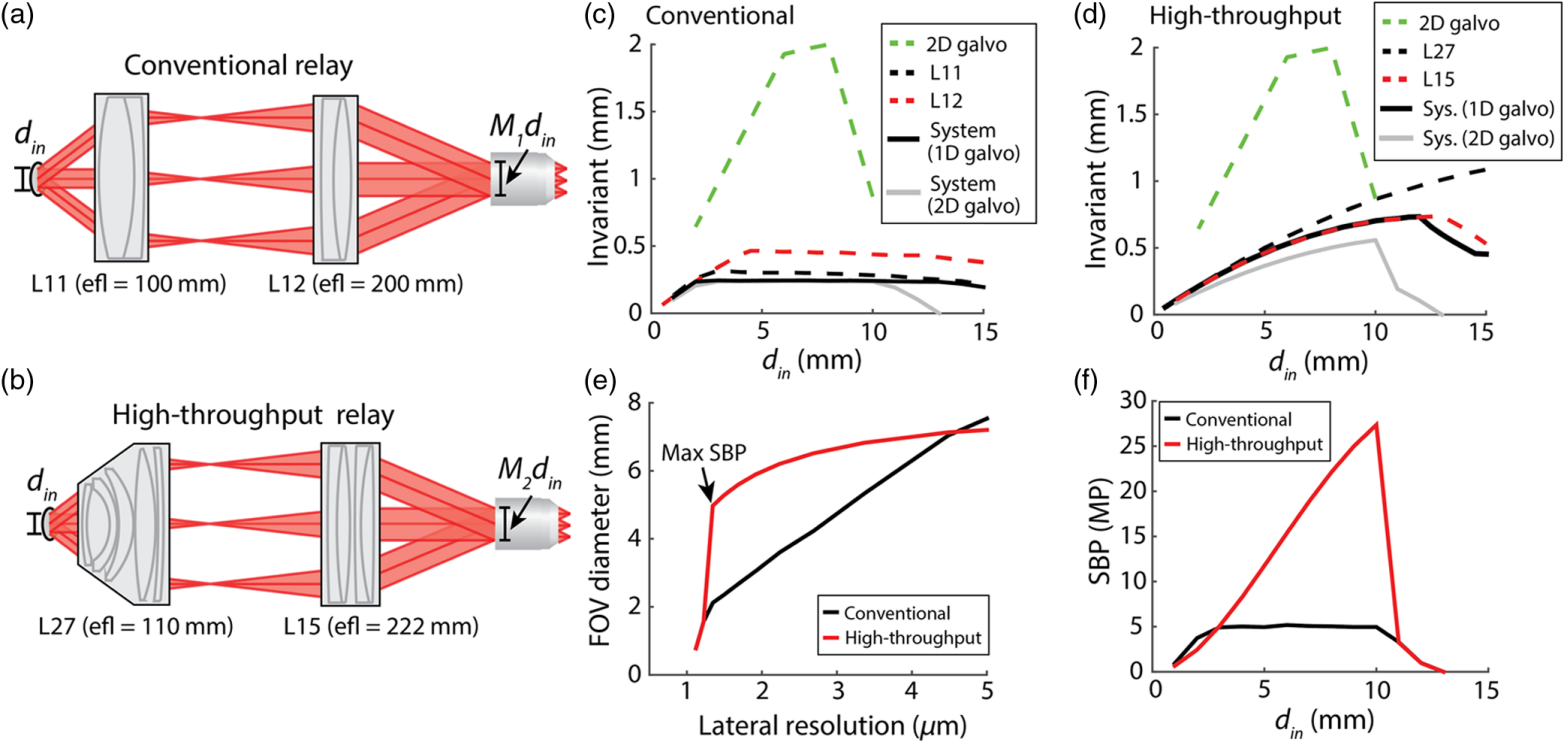
Comparison of integrated scanning systems for conventional TPM and LF-TPM. (a) Schematic of conventional scanning system consisting of two achromatic doublets with effective focal length equal to 100 and 200 mm (L11 and L12 in Appendix B). The input beam diameter $d_{\text{in}}$ is magnified by a factor of $M_{1}$ at the output of the relay. (b) Schematic of high-throughput relay with lenses highlighted in Fig. 5 (L27 and L15). The magnification of the relays in (a) and (b) ($M_{1}$ and $M_{2}$) is approximately equal to 2. (c) Simulated performance of conventional relay lenses with objective modeled as paraxial lens with a focal length of 45 mm in Zemax. Optical invariant of isolated components (2-D galvanometer, L11, and L12) and integrated relay with 1-D and 2-D galvanometer are plotted as a function of input beam diameter to the system $d_{\text{in}}$. (d) Same as (c), but for the high-throughput relay lenses shown in (b). (e) The FOV predicted for the two systems plotted as a function of lateral resolution. FOV and lateral resolution were calculated as a function of $d_{\text{in}}$ using Eqs. (5), (9), and (17). The maximum SBP is achieved for input beam diameter equal to 10 mm. (f) SBP predicted for the two systems plotted as a function of $d_{\text{in}}$ (Eq. 10).

Once candidate components were analyzed in isolation, it was necessary to evaluate the performance of the integrated microscope, which requires consideration of the magnification of the relay and the corresponding operating conditions of the relay lenses and scan mirrors. Consider a microscope with two lenses that relay the input beam onto the rear aperture of the objective [Fig. 6(a)]. The beam diameter at the rear of the objective will be

$$d_{\text{out}}=\frac{f_{2}}{f_{1}}d_{\text{in}}=M_{r}d_{\text{in}},$$ (16)

where $d_{\text{out}}$ is the beam diameter at the rear of the objective, $f_{2}$ is the focal length of the second relay lens, $f_{1}$ is the focal length of the first relay lens, $d_{\text{in}}$ is the input beam diameter, and $M_{r}$ is the magnification of the relay. If $d_{\text{out}}$ is less than the pupil diameter of the objective, then the objective is underfilled and will not excite the sample with the full NA of the objective. In this case, the underfilled objective has an effective excitation NA defined as

$${\mathrm{NA}}_{ex}=\frac{d_{\text{out}}}{d_{\text{pupil}}}\mathrm{NA},$$ (17)

where ${\mathrm{NA}}_{ex}$ is the excitation NA, $d_{\text{pupil}}$ is the diameter of the objective pupil defined in Eq. (7), and NA is the full NA of the objective lens.

We modeled the performance of two microscopy systems both with relay magnification equal to 2 using Zemax: a conventional TPM system consisting of achromatic doublets L11 and L12 [Fig. 6(a)], and a high-throughput microscope consisting of L27 and L15 [Fig. 6(b)]. The relays were analyzed with either a 1-D or 2-D galvanometer mirror system (GVS012, Thorlabs). The 2-D galvanometer mirrors were modeled with two mirrors reflecting the beam in orthogonal directions separated by 25.7 mm. When modeling with the 2-D galvanometer, the front focal plane of the first relay lens was positioned between the two mirrors, as suggested by manufacturers. An objective lens modeled as a paraxial lens with a focal length of 45 mm was then positioned at the output of the relays.^16^ Similar to the isolated mirror scanners and relay lenses, the performance of these integrated systems was evaluated over a range of input beam diameters from 1 to 15 mm. The optical invariant of the integrated system at a given input beam diameter was calculated by determining the maximum scan angle before the beam was clipped or the spot was no longer diffraction limited at the specimen plane. The excitation NA of the objective in this model depends on the diameter of the beam at the rear aperture as defined by Eq. (17).

From this analysis, we calculated the optical invariant as a function of $d_{\text{in}}$ for the conventional TPM system [Fig. 6(c)] and the high-throughput system [Fig. 6(d)]. Also included on these plots is the optical invariant of the isolated components in the system (e.g., relay lenses and 2-D galvanometer mirrors) plotted with respect to the input beam diameter of the integrated microscope, which can help identify the limiting component and optimal imaging condition for the integrated system. We also calculated the expected FOV that could be imaged as a function of the lateral resolution of the systems by plugging the effective excitation NA of the objective lens into Eq. (9) [Fig. 6(e)]. The SBP was also plotted as a function of $d_{\text{in}}$ for both systems [Fig. 6(f)]. The microscope’s information transmission is maximum at an input beam diameter of around 10 mm (i.e., excitation NA of 0.22) for the high-throughput system. Although the high-throughput system does not fully support the macro-objective lenses, our simulations predict that this system will increase the SBP by approximately fivefold in comparison with conventional relay lenses.

Until this stage of the design, we have focused on selecting potential relay lenses for LF-TPM and evaluating their performance together in a single relay with a 2-D galvanometer. The microscope can also be designed with two scanners separated by another afocal relay. Both a two-relay system with scan mirrors separated by an afocal relay and a single-relay system with a 2-D galvanometer have their advantages and disadvantages. Any addition of relay lenses into a microscope will increase the complexity and make it more difficult to minimize optical aberrations. On the other hand, a 2-D galvanometer system may introduce aberrations and/or vignetting at large scan angles and thus decrease the scannable FOV of the microscope, as shown in our simulations. Due to the fact that it is more challenging and expensive to construct a microscope with two relays, we opted to implement a LF-TPM system with a single relay consisting of L15, L27, and a 2-D galvanometer (GVS012, Thorlabs).

### 2.6 Collection Optics

The collection system was designed by considering the photocathode of the PMT at a plane conjugate to the specimen plane. In our design, the emission NA is the full NA of the objective lens, not the lower excitation NA. Therefore, we modeled potential collection systems with light exiting an aperture with a diameter of 25.2 mm at an angle of 4.5 deg and a photocathode with an 8-mm diameter (R12829, Hamamatsu, Hamamatsu City, Japan). Using Eq. (3), we predicted the collection system to require an NA>0.23 [$I=12.6\;\sin(4.2\;\deg)=0.92\;\mathrm{mm}\to {\mathrm{NA}}_{\mathrm{coll}}\geq 0.92/4=0.23$]. The system was designed with three plano-convex lenses, which are listed in Appendix C, and tested using Zemax. The photocathode is positioned between the image plane and exit pupil of the collection system, not directly at the image plane. There is no expected vignetting of the emission for imaging conditions without scattering when using the Olympus XLFLUOR4X and only 14% clipped rays when imaging off-axis with the MVPLAPO 2XC objective.

### 2.7 System Overview

**Fig. 7:**
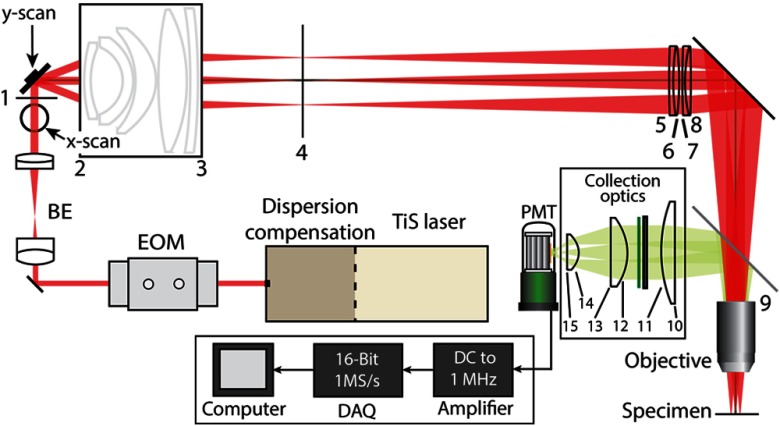
LF-TPM system schematic. Pulsed light from TiSapphire (TiS) laser is directed to the input of the microscope. Laser intensity and dispersion are controlled with an electro-optic modulator (EOM) and dispersion compension prisms. The beam is then expanded with a beam expander (BE) consisting of two achromatic doublets. Emission is separated with a dichroic mirror and is transmitted through both a shortpass and notch filter before being collected by a photomultiplier tube (PMT). The output of the PMT is amplified and digitized before images are displayed on a computer. Surfaces are labeled with numbers, and the distances are presented in Appendix C.

The final LF-TPM system includes a TiSapphire laser (Mai Tai HPDS, Spectra Physics, Santa Clara) with an electro-optic modulator (350-80-LA-02, Conoptics, Danbury) and prism compensation for controlling the laser intensity and dispersion at the sample, respectively (Fig. 7). To achieve the maximum SBP of the system, the beam is expanded to 10-mm beam diameter before the galvanometer using two achromatic doublets. We also tested the performance of the system with an input beam diameter of 5 mm using a variable beam expander. Before the PMT, the emission from the sample is separated with a dichroic mirror (FF775-Di01-60 ×84, Semrock, Rochester) and emission filters (FF01-680/SP-50.8-D, Semrock and ET525/36 m, Chroma, Foothill Ranch). The signal is then amplified with a transimpedance amplifier (TIA60, Thorlabs), low-pass filtered (≤500 kHz passband, EF506, Thorlabs), and digitized (PCIe-6353, National Instruments, Austin) before collection by a computer. Control of the electronics and image construction are then performed using custom-written software (MATLAB, MathWorks, Natick). All the part and component distances in the microscope are listed in Appendix C.

## 3 Experimental Results

### 3.1 Experimental Validation with Fluorescein and Fluorescent Microspheres

**Fig. 8:**
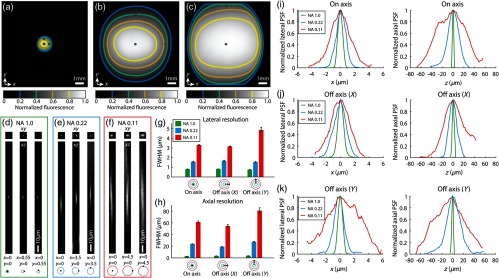
Experimental FOV and resolution measurements. (a) Normalized fluorescence signal measured across the FOV using the high-throughput relay shown in Fig. 6–7 and the Olympus XLUMPLFLN (20X, NA 1.0). (b) Same as (a), but for Olympus XLFLUOR4X with rear aperture underfilled to an effective NA of 0.22 (10 mm input beam diameter to microscope). (c) Same as (a)-(b), but for Olympus XLFLUOR4X with rear aperture underfilled to an effective NA of 0.11 (5 mm input beam diameter to microscope). (d) Lateral and axial cross section of PSF measured experimentally by imaging 0.5-μm diameter fluorescent beads embedded in agarose. Results are for high-throughput relay and NA 1.0 (Olympus XLUMPLFLN). Beads were imaged both on and off axis as specified underneath cross sections. (e) Same as (d), but with the Olympus XLFLUOR4X and effective NA of 0.22. (f) Same as (d)-(e), but with rear aperture underfilled to an effective NA of 0.11. (g) Estimation of lateral resolution measured as FWHM of PSF shown in (d)-(f). (h) Same as (g), but for axial resolution. (i) Profile of lateral and axial PSF for beads imaged on axis shown in (d)–(f). (j) Same as (i), but for beads imaged off axis in the x-direction. (k) Same as (i), but for beads imaged off axis in the y-direction.

We tested the performance of our system experimentally by placing fluorescein at the sample plane and measuring the fluorescence signal over the FOV with the Olympus XLFLUOR4X underfilled to either NA 0.22 (i.e., input beam diameter to galvo of 10 mm) or NA 0.11 (i.e., input beam diameter to galvo of 5 mm) [Figs. 8(b) and 8(c)]. For reference, we also measured the fluorescence signal using a 20× objective with the same high-throughput relay system [Fig. 8(a)]. The fluorescence signal remained above 0.4 times the maximum over FOV diameters of 1.4, 7, and 9 mm with the Olympus XLUMPLFLN (20×, NA 1.0), Olympus XLFLUOR4X underfilled to NA 0.22, and XLFLUOR4X underfilled to NA 0.11, respectively.

The resolution was measured over the FOV by imaging 0.5-μm fluorescent microspheres [18859-1, Polysciences, Warrington; Figs. 8(d)–8(h)] embedded in a thick agarose gel. The FWHM, lateral profiles, and axial profiles of the PSF are also shown in [Figs. 8(d)–8(k)]. As expected, underfilling the objective enables larger field imaging but worsens the resolution, especially axially in comparison with the Olympus XLUMPLFLN. However, our optimal design (XLFLUOR4X underfilled to NA 0.22) can achieve a SBP of 35MP, which is 3.2 times more than what can be achieved with a conventional TPM system (Table 1). The lateral and axial resolutions of the LF-TPM system over the 7-mm-diameter FOV are <1.7 and <28  μm, respectively. This demonstrates the performance and range of imaging fields achievable with these relay lenses, galvanometer, and objective.

**Table 1:** Imaging capabilities of isolated objectives, conventional TPM, and LF-TPM. Lateral resolution for conventional TPM with pixel averaging is effective resolution, not optical resolution. For LF-TPM, the theoretical and experimental lateral resolution are included.

Objective or system	NA	Lateral resolution (μm)	FOV diameter (Ø mm)	Rectangular FOV (mm×mm)	Pixels for rectangular FOV	FOV area (${\mathrm{mm}}^{2}$)	SBP (MP)
Olympus XLFLUOR4X	0.28	1.08	6.63	4.69×4.69	8709×8709	21.98	75.85
Olympus XLUMPLFLN 20X	1	0.31	1.10	0.78×0.78	5081×5081	0.61	25.82
Conventional TPM	1	0.31	0.71	0.50×0.50	3280×3280	0.25	10.76
Conventional TPM (pixel averaging)	1	1.96	0.71	0.50×0.50	512×512	0.25	0.26
LF-TPM system	0.22	1.37/1.68	7.00	4.95×4.95	5893×5893	24.50	34.72

For completeness, we also conducted fluorescein and microsphere imaging with the Olympus MVPLAPO 2XC but measured worse resolution off axis than what was achieved with the Olympus XLFLUOR4X (Appendix D).

### 3.2 In Vivo Applications of LF-TPM: Imaging the Cerebral Vasculature and Microglia Cell Bodies

**Fig. 9:**
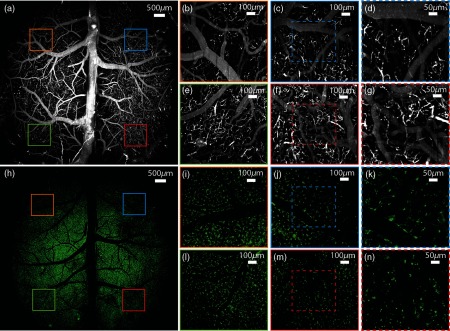
Cerebral vasculature and microglia imaged over the mouse cortex with LF-TPM. (a) Maximum projection image of cerebral vessels imaged with LF-TPM after tail vein injection of fluorescein-dextran. Dimensions of the box are $8\times 8\;{\mathrm{mm}}^{2}$. (b) $1\times 1\;{\mathrm{mm}}^{2}$ FOV imaged 3-mm off axis at orange box shown in (a). (c) $1\times 1\;{\mathrm{mm}}^{2}$ FOV imaged 3-mm off axis at blue box in (a). (d) $500\times 500\;{\mu m}^{2}$ FOV highlighted by dashed blue box in (c). (e) $1\times 1\;{\mathrm{mm}}^{2}$ FOV imaged 3-mm off axis at green box shown in (a). (f) $1\times 1\;{\mathrm{mm}}^{2}$ FOV imaged 3-mm off axis at red box shown in (a). (g) $500\times 500\;{\mu m}^{2}$ FOV highlighted by dashed red box in (f). (h)–(n) Same as (a)–(g), but for microglia imaged in $\mathrm{Cx}3\mathrm{Cr}1^{\mathrm{GFP}+/-}\mathrm{mice}$.

After experimental validation of the system, we performed *in vivo* imaging of the mouse cerebral vasculature and microglia (Fig. 9). To image the cerebral micro-architecture, we removed an ∼9-mm-diameter portion of the mouse skull.^29^^,^^30^ The full surgical procedure is described in Appendix E. We then imaged the cerebral vasculature in male C57BL6 mice after tail vein injection of fluorescein-dextran and the microglia in mice with GFP knocked-in to the Cx3Cr1 locus ($\mathrm{Cx}3\mathrm{Cr}1^{\mathrm{GFP}+/-}$).

To maximize the information transmitted by our system, we imaged the mouse cortex under optimal system conditions (i.e., XLFLUOR4X underfilled to NA 0.22, SBP approximately 35MP). Due to the large relatively flat field and axial sectioning of the microscope, the curvature of the mouse brain poses a challenge: the image plane is not perpendicular to the surface of the mouse brain over the FOV. Thus, fluorescence signal measured in a single frame is only over an elliptic region of the brain that depends on how the objective front focal plane intersects with the mouse brain. To image over the entire FOV, the brain was scanned axially by moving the mouse on a motorized stage (MLJ050, Thorlabs). Each image was scanned at a 50-Hz line rate. Most of the images were scanned with 1000 lines for a slice acquisition time of ∼20  s. The translation time between axial positions was ∼1  s and therefore did not contribute significantly to total acquisition time. To demonstrate the capabilities of the system, we scanned both low-resolution scans of the full FOV [Figs. 9(a) and 9(h); $8\times 8\;{\mathrm{mm}}^{2}$, 1000×1000  pixels] and high-resolution scans of smaller fields 3 mm off axis [Figs. 9(b)–9(e) and 9(i)–9(l); $1\times 1\;{\mathrm{mm}}^{2}$, 1000×1000  pixels]. Also included are images with a FOV similar to conventional TPM [Figs. 9(f) and 9(g) and 9(m)–9(n); $500\times 500\;{\mu m}^{2}$, 500×500  pixels]. For all imaging, the mouse remained in the same lateral (x,y) position relative to the objective without tracking motion.

We also calculated the FWHM of capillary vessel diameters to determine resolution capabilities of our system for *in vivo* applications. The system was able to image vessel diameters as small as 3  μm over the entire FOV, as well as ∼22,500 microglia with a cell body diameter of ∼5  μm over the cortex of $\mathrm{Cx}3\mathrm{Cr}1^{\mathrm{GFP}+/-}\mathrm{mice}$.

## 4 Discussion and Conclusion

Both conventional TPM and MOIPI have improved our understanding of the functional architecture of the mouse cortex. However, both of these imaging modalities are limited by the trade-off between resolution and FOV. In this report, we have shown the potential that LF-TPM has in studying the mouse brain over multiple spatial scales. Our results demonstrate almost a 100-fold increase in the FOV area and 3.2-fold increase in information transmission in comparison with conventional TPM, all while maintaining <1.7-μm lateral and <28-μm axial resolution (Table 1). Here, we highlighted *in vivo* imaging of the cerebral vasculature and microglia cell bodies over a 7-mm-diameter FOV. In addition to improving our understanding of the cellular and vascular mechanisms underpinning resting-state functional brain connectivity,^3^ LF-TPM may also improve our understanding of the cellular dynamics of other neural phenomena that occur over large regions of the mouse cortex, such as cortical spreading depression,^31^ retinal waves,^32^ and Mayer waves.^33^ Imaging individual microglia cells over such a large portion of the cerebral vasculature also has the potential for fundamental discoveries in the inflammatory responses that occur in stroke, multiple sclerosis, and neurodegenerative diseases.^34^^–^^36^

In comparison with other groups that have extended the FOV in TPM by custom designing each component, our design depends on a simple optical invariant framework, first to evaluate potential lenses and second to design scan relays suitable for large FOV imaging.^14^^,^^15^ This approach permits the microscope designer to isolate components and compare their performance with the throughput demands of the objective lens free from the complexities of a fully integrated microscope design. As a result, it can be easily adapted to increase the FOV in any other custom-built laser-scanning microscope, making it ideal for groups searching for cost-effective large FOV microscopes constructed with off-the-shelf components.

One of the limitations of analyzing the optical invariant function of isolated components is that it may not account for additive aberrations introduced when integrating multiple optical components. Therefore, it may be necessary to identify several potential relay lens candidates and test multiple combinations of relays. Other LF-TPM designs consist of custom-designed optics that may have lower throughput in isolation but compensate for aberrations introduced by other optics in the system, such as the remote focusing objective designed by Sofroniew et al.^14^ and the scan compensation lenses implemented by Tsai et al.^16^ In cases such as these, the design strategy in this report can be applied, but it requires analyzing subsystems with counteracting aberrations together. There may also be other optimization parameters in addition to throughput, such as propagation time delay difference, that could improve the signal-to-noise ratio (SNR) of the system, which were overlooked in the analysis described here.^14^ Despite these limitations in this design approach, the primary drawback is the lack of commercially available relay lenses that match the demands of high-throughput objectives, as testament to our difficulty in identifying a high-throughput, long focal length tube lens.

**Fig. 10:**
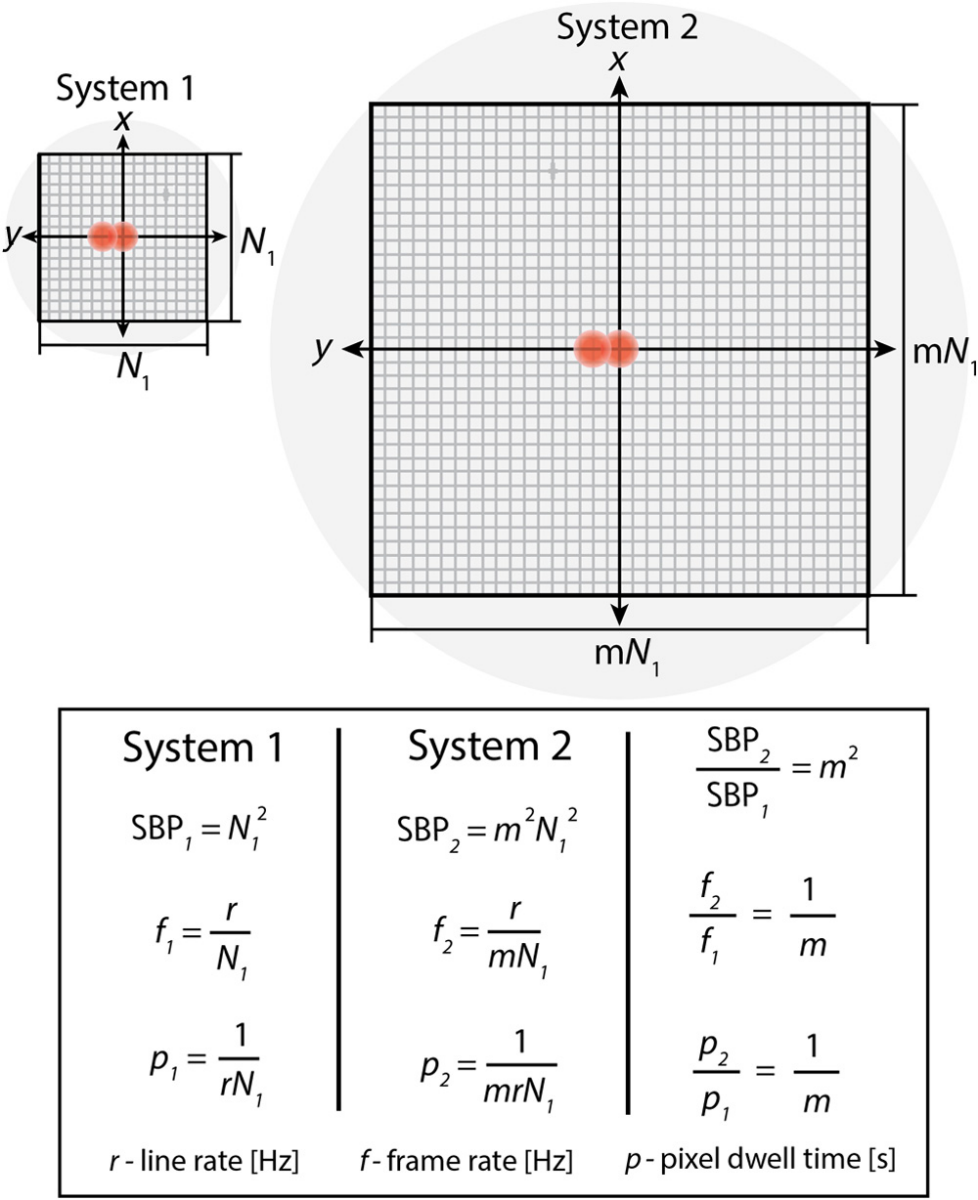
Differences in frame rate and pixel dwell time for systems with different SBP. System 1 resolution and FOV require $N_{1}$ lines to sufficiently sample FOV. System 2 resolution and FOV require a factor of m more lines to be scanned. The resulting frame rate f of the systems that results from imaging the full SBP is equal to the line rate r divided by the number of scanned lines. Despite increased SBP, system 2 frame rate decreases by a factor of m. The pixel dwell time p for system 2 also decreases by a factor of m in comparison with system 1.

Separate from the optical invariant design approach, our current system has two potential shortcomings: imaging speed and an anisotropic PSF in the axial and lateral dimensions. Due to the raster scanning necessary to acquire images, a major challenge in applying LF-TPM to functional brain imaging is imaging speed and SNR. If the SBP of an LF-TPM system requires scanning m more lines per frame in comparison with conventional TPM, then the frame rate and pixel dwell time will each decrease by a factor of m (Fig. 10). Fortunately, traditional galvanometer mirrors have the advantage of flexible scanning patterns that enable imaging multiple subregions or unique scan geometries within the FOV.^37^^,^^38^ Another option for increasing the frame rate and SNR is to simply scan the entire FOV with fewer lines. This is already commonly done in conventional TPM systems, which typically undersample images by a factor of around 3 to 5 to collect 512×512  pixel images at frame rates of 1 Hz with traditional galvanometers or 30 Hz using resonant scanners.^1^^,^^2^^,^^39^ The effective lateral resolution in such an imaging paradigm is around 2  μm, which is actually larger than the capabilities of our system (Table 1). If the SNR of our LF-TPM system is sufficient for an application, then the limiting factor to the imaging speed is the scan rate of the galvanometer mirrors. We opted to reduce the complexity of our system using a single relay and thus are limited to imaging at ∼100  lines/s. Multifocal TPM or other PSF engineering techniques may also serve to improve the image acquisition rate in LF-TPM.^12^^,^^13^^,^^40^

Because the axial resolution is inversely proportional to NA squared, the PSF is stretched in the axial direction. The anisotropic PSF is probably also due to aberrations introduced by the relay lenses, the 2-D galvanometer, and/or the objective lens itself. Although functional measurements of individual cells may be confounded by a PSF that stretches beyond subcellular resolution in the axial direction, an ellipsoidal-shaped PSF may also be advantageous depending on the imaging conditions.^21^ Indeed, the Bessel beam has been utilized for live sample imaging of neurons to extend the depth of field and increase volumetric imaging rates.^41^^,^^42^

Regardless of these challenges, LF-TPM holds great promise for *in vivo* imaging of the mouse cortex. Here, we have presented an intuitive design approach for developing LF-TPM with off-the-shelf components. As highlighted by our modeling and experimental results, analysis of the optical invariant can lead to lower cost LF-TPM designs, as well as minimize the need for custom-designing relay lenses using optical engineering software.

## Figures and Tables

**Fig. 11:**
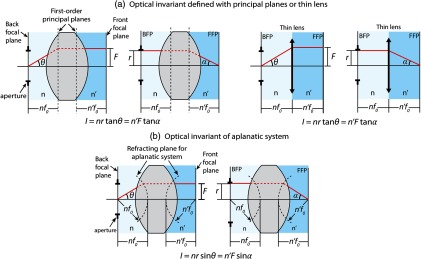
Definition of optical invariant. (a) Optical system with effective focal length $f_{0}$ modeled with first-order principal planes. The refractive indices in image and object space are labeled as n and $n^{\prime }$, respectively. Front and rear focal lengths depend on the refractive indices. Back focal plane and aperture stop coincide. Marginal and chief rays are shown to visualize FOV, NA, and optical invariant. Also shown is the model of the same system with a thin lens. (b) Same as (a), but for aplanatic model. Refracting surfaces are spherical. This definition is also known as the Abbe sine condition.

**Fig. 12:**
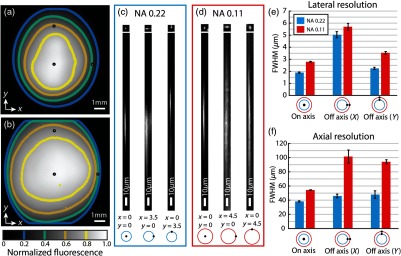
Experimental FOV and resolution measurements for MVPLAPO 2XC. (a) Normalized fluorescence measured with high-throughput relay shown in Figs. 6 and 7 and the MVPLAPO 2XC with rear aperture underfilled to an effective NA of 0.22. Result is for 10-mm input beam diameter. (b) Same as (a), but for MVPLAPO 2XC with rear aperture underfilled to an effective NA of 0.11. (c) Lateral and axial cross section of PSF measured experimentally by imaging 0.5-μm-diameter fluorescent beads embedded in agarose. Results are for high-throughput relay and MVPLAPO 2XC with rear aperture underfilled to an effective NA of 0.22. Beads were imaged both on and off axis as specified underneath cross sections (d) Same as (c), but with an MVPLAPO 2XC with rear aperture underfilled to an effective NA of 0.11. (e) Estimation of lateral resolution measured as FWHM of PSF shown in (c)–(d). (f) Same as (e), but for axial resolution.

## Appendix A: Definition of Optical Invariant

The optical invariant can be defined in several ways. For this report, we were interested in analyzing diffraction-limited microscopy systems. If the optical system or component is modeled with first-order principal planes, then the invariant at the aperture and field planes is defined as

$$I=nr\;\tan\;\theta =n^{\prime }F\;\tan\;\alpha ,$$ (18)

where I is the optical invariant, n and $n^{\prime }$ are the indices of refraction of the media before and after the optical component, respectively, r and θ are the beam radius and incident angle of collimated light at the aperture plane, respectively, and F and α are the FOV radius and angle of the cone of light at the image plane, respectively [Fig. 11(a)].^23^ For paraxial rays,^22^ Eq. (18) simplifies to

$$I=nr\theta =n^{\prime }F\alpha .$$ (19)

Although Eq. (18) is valid for systems modeled with thin lenses and Eq. (19) is valid for paraxial systems, microscopy systems are more accurately modeled with spherical refracting surfaces centered at the front and back focal points of the optical system [Fig. 11(b)]. The vertices of these spherical surfaces are located at the principal points of the optical system. In this case, the optical invariant is defined as

$$I=nr\;\sin\;\theta =n^{\prime }F\;\sin\;\alpha .$$ (20)

Equation (20) is also known as the Abbe sine condition, which is satisfied for aplanatic imaging systems, such as a diffraction-limited microscope.^24^ Thus, we opted to use Eq. (20) to define the optical invariant. This definition served as a useful guide for assessing the optical throughput of the components even in cases for lenses that were not aplanatic or for f-theta telecentric scan lenses for which F=fθ, where f is the focal length of the scan lens.

## Appendix B: Components Analyzed for LF-TPM

### B.1 Objective Lenses

We analyzed 45 commercially available Olympus objective lenses in this report (Table 2). The invariant and SBP product were calculated using Eqs. (8) and (10), respectively.

**Table 2:** Specifications of 45 commercially available Olympus objective lenses analyzed.

Objective	M	FN	NA	n	Pupil dia. (mm)	Invariant (mm)	SBP (MP)
UPLSAPO 4X	4	26.5	0.16	1	14.4	0.530	24.7
UPLFLN 4X	4	26.5	0.13	1	11.7	0.431	16.3
UPLFLN 4XP	4	26.5	0.13	1	11.7	0.431	16.3
PLN 4X	4	22	0.1	1	9	0.275	6.7
MVPLAPO 2 XC	4	22	0.5	1	45	1.375	166.5
XLFLUOR4X/340	4	26.5	0.28	1	25.2	0.928	75.7
UPLSAPO 10X2	10	26.5	0.4	1	14.4	0.530	24.7
UPLFLN 10X2	10	26.5	0.3	1	10.8	0.398	13.9
UMPLFN 10XW	10	26.5	0.3	1.33	10.8	0.398	13.9
UPLFLN 10XP	10	26.5	0.3	1	10.8	0.398	13.9
PLN 10X	10	22	0.25	1	9	0.275	6.7
XLPLN10XSVMP	10	18	0.6	1.33	21.6	0.540	25.7
UPLSAPO 20X	20	26.5	0.75	1	13.5	0.497	22.2
UPLFLN 20X	20	26.5	0.5	1	9	0.331	9.7
XLUMPLFLN-W	20	22	1	1.33	18	0.550	25.8
UMPLFN 20XW	20	26.5	0.5	1.33	9	0.331	9.7
UPLFLN 20XP	20	26.5	0.5	1	9	0.331	9.7
UCPLFLN 20X	20	22	0.7	1	12.6	0.385	13.1
LUCPLFLN 20X	20	22	0.45	1	8.1	0.248	5.4
LUCPLFLN 40X	20	22	0.6	1	10.8	0.330	9.6
PLN 20X	20	22	0.4	1	7.2	0.220	4.3
UAPON 20XW340	20	22	0.7	1.33	12.6	0.385	13.1
XLPLN25XWMP2	25	18	1.05	1.33	15.12	0.378	12.1
XLPLN25XSVMP2	25	18	1	1.33	14.4	0.360	11.1
XLSLPLN25XSVMP2	25	18	0.95	1.33	13.68	0.342	10.1
XLSLPLN25XGMP	25	18	1	1.41	14.4	0.360	11.1
UPLSAPO 30XS	30	22	1.05	1.41	12.6	0.385	12.5
UPLSAPO 60XO	40	26.5	0.95	1	8.55	0.315	8.5
UPLFLN 40X	40	26.5	0.75	1	6.75	0.248	5.5
UPLFLN 40XO	40	26.5	1.3	1.51	11.7	0.431	15.1
LUMPLFLN 40XW	40	26.5	0.8	1.33	7.2	0.265	6.2
UPLFLN 40XP	40	26.5	0.75	1	6.75	0.248	5.5
PLN 40X	40	22	0.65	1	5.85	0.179	2.8
UAPON 40XWO340-2	40	22	1.35	1.51	12.15	0.371	11.1
UAPON 40XW340	40	22	1.15	1.33	10.35	0.316	8.3
UPLSAPO 40X3	60	26.5	1.35	1.51	8.1	0.298	7.2
PLAPON 60XO	60	26.5	1.42	1.51	8.52	0.314	7.9
UPLFLN 60X	60	26.5	0.9	1	5.4	0.199	3.4
LUMPLFLN 60XW	60	26.5	1	1.33	6	0.221	4.2
LUMFLN 60XW	60	26.5	1.1	1.33	6.6	0.243	5.0
LUCPLFLN 60X	60	22	0.7	1	4.2	0.128	1.5
UPLSAPO 100XO	100	26.5	1.4	1.51	5.04	0.186	2.8
UPLFLN 100XO2	100	26.5	1.3	1.51	4.68	0.172	2.4
UPLFLN 100XOP	100	26.5	1.3	1.51	4.68	0.172	2.4
PLN 100XO	100	22	1.25	1.51	4.5	0.138	1.6

### B.2 Mirror Scanners

The commercially available resonant scanners, galvanometer mirrors, and polygonal mirror scanners analyzed in the report are listed in Table 3.

**Table 3:** Commercially available laser scanners analyzed. The line rate for polygonal scanners corresponds to a polygon rotation rate of 10,000 rotations per minute.

Scanners			
Component	Part number	Vendor	Line rate (kHz)
Galvanometer scanner (1D)	GVS011	Thorlabs	0.13 (square) and 0.26 (sine)
Galvanometer scanner (2-D)	GVS012	Thorlabs	0.13 (square) and 0.26 (sine)
Resonant scanner 4 kHz	CRS4K	Cambridge Technologies	8 (sine)
Resonant scanner 8 kHz	CRS8K	Cambridge Technologies	16 (sine)
Resonant scanner 12 kHz	CRS12K	Cambridge Technologies	24 (sine)
18 facet polygon mirror scanner	DT-18-275-040	Lincoln Laser	3 (unidirectional saw)
36 facet polygon mirror scanner	DT-36-275-040	Lincoln Laser	6 (unidirectional saw)
54 facet polygon mirror scanner	DT-54-275-040	Lincoln Laser	9 (unidirectional saw)
72 facet polygon mirror scanner	DT-72-275-040	Lincoln Laser	12 (unidirectional saw)

### B.3 Relay Lenses

To calculate the max invariant as a function of input beam diameter for a relay lens, we first modeled the lens in telecentric configuration using OpticStudio. The spot size over the image plane was calculated for input beam diameters ranging from 1 to 30 mm and scan angles ranging from 0 deg to 25 deg. We adjusted the image plane field curvature to minimize spot size, which enabled us to distinguish spot size increases due to defocus from spot size increases due to astigmatism, coma, and spherical aberrations. Using OpticStudio, we were able to compare the spot RMS radius to the Airy radius over the FOV. The resolution was considered to be limited by optical aberrations, not diffraction, when the spot RMS radius was greater than the Airy radius. Vignetting data were also recorded to determine the angle at which the beam was <100% transmitted by the system, ignoring absorption and reflection of light through the system. To determine the maximum scan angle for a given input beam diameter, the max angle before the beam was clipped was compared with the scan angle at which the spot RMS radius was greater than the Airy radius. Depending on the imaging conditions and lens, the maximum scan angle would be limited by either vignetting or optical aberrations (Table 4).

**Table 4:** Step-by-step procedure for calculating the invariant function for relay optics.

Zemax	
Step	Description
1	Pick a test optic
2	Model in telecentric configuration
3	Set input beam diameter to 1 mm
4	Set fields ranging from 0 deg to field that beam is clipped by 10%
5	Constrain image surface to paraxial focus
6	Save field curvature and distortion as .txt file
7	Set curvature of image surface to minimize spot size
8	Create merit function to minimize spot size error at image plane
9	Set last surface distance to variable
10	Optimize
11	Record Airy radius for this input beam diameter
12	Record RMS spot size over field
13	Record percent of unvignetted rays over field
14	Increase beam radius by 0.5 mm
15	Repeat step #10 to 14 until vignetting at every field or spot is limited by optical aberrations at every field
Matlab	
Step	Description
1	Set input beam radius for analysis
2	Extract spot size, vignetting, Airy radius, and field curvature data from .txt files
3	Find angle that beam is clipped
4	Find max angle that beam is diffraction limited at image plane
5	Compare angles from steps 3 and 4 to find max scan angle for given input beam diameter
6	Calculate product of max beam angle and radius
7	Increase beam radius
8	Repeat steps #1 to 7 until max beam radius is reached
9	Plot invariant versus input beam radius

The 27 relay lenses that we analyzed in this report are included in Table 5. We included relay lenses that were used in other LF-TPM systems.^14^^,^^15^ However, due to compensation optics that may be used in these systems, the performance of the relay lens in isolation may not be representative of the throughput of the integrated microscope.

**Table 5:** Specifications of relay lenses analyzed.

Lens ID#	Type	Focal length (mm)	Wavelength (nm)	Vendor	Part number
1	Plano-convex	50	600 to 1050 (coating only)	Edmund Optics	48-795
2	Plano-convex	60	725 to 1050 (coating only)	Qioptiq	G312340000
3	Plano-convex	60	650 to 1050 (coating only)	Thorlabs	LA1401-B
4	Plano-convex	100	600 to 1050 (coating only)	Edmund Optics	48-797
5	Plano-convex	100	725 to 1050 (coating only)	Qioptiq	G312334000
6	Plano-convex	100	650 to 1050 (coating only)	Thorlabs	LA1050-B
7	Plano-convex	200	600 to 1050 (coating only)	Edmund Optics	48-801
8	Plano-convex	200	650 to 1050 (coating only)	Thorlabs	LA1979-B
9	Plano-convex	300	725 to 1050 (coating only)	Qioptiq	G312363000
10	NIR achromatic doublet	100	750 to 1100	Edmund Optics	47-317
11	NIR Achromatic doublet	100	650 to 1050	Thorlabs	AC508-100-B
12	NIR achromatic doublet	200	650 to 1050	Thorlabs	AC508-200-B
13	Compound achromatic doublet	100	650 to 1050	Thorlabs	AC508-200-B and AC508-200-B
14	Compound achromatic doublet	150	488 to 514	Qioptiq	322278000 and 322278000
15	Compound achromatic doublet	222	650 to 1050	Thorlabs	AC508-400-B and AC508-500-B
16	Compound achromatic doublet	222	650 to 1050	Thorlabs	AC508-400-B and AC508-500-B (flipped)
17	Compound achromatic doublet	245	488 to 514	Qioptiq	322278000 and 322242000
18	Compound achromatic doublet	500	650 to 1050	Thorlabs	AC508-1000-B and AC508-1000-B
19	Telecentric f-theta scan lens	18	810 to 890 and 1000 to 1100	Thorlabs	LSM02-BB
20	Telecentric f-theta scan lens	36	810 to 890 and 1000 to 1100	Thorlabs	LSM03-BB
21	Telecentric f-theta scan lens	54	810 to 890 and 1000 to 1100	Thorlabs	LSM04-BB
22	Telecentric f-theta scan lens	54	750 to 950	Thorlabs	LSM54-850
23	Telecentric f-theta scan lens	58	920	Various	N/A
24	Telecentric f-theta scan lens	100	633	Edmund Optics	64-426
25	Telecentric f-theta scan lens	100	633	Various	64-426
26	Telecentric f-theta scan lens	110	810 to 890 and 1000 to 1100	Thorlabs	LSM05-BB
27	Telecentric f-theta scan lens	115	1064	Edmund Optics	64-422
28	Telecentric f-theta scan lens	235	920	Various	N/A
29	Plano-convex	50	600 to 1050 (coating only)	Edmund Optics	48-795

## Appendix C: System Prescription

The distances between components in the system are listed in Table 6.

**Table 6:** System prescription starting from 2-D galvanometer mirrors to PMT. Surface location is labeled in Fig. 7.

Surfaces	Distance or thickness (mm)	Description	Part	Vendor
1	—	XY galvanometer	GVS012	Thorlabs
1 to 2	38	—	—	—
2 to 3	92	Telecentric f-theta scan lens	64-422	Edmund optics
3 to 4	348	—	—	—
5 to 6	7.1	Achromatic doublet	AC508-500-B	Thorlabs
6 to 7	2	—	—	—
7 to 8	7.1	Achromatic doublet	AC508-400-B	Thorlabs
8 to 9	208	—	—	—
9	—	Rear aperture	XLFLUOR4X	Olympus
9 to 10	100	—	—	—
10 to 11	10.1	Collection lens 1	LA1353-A	Thorlabs
11 to 12	25	—	—	—
12 to 13	12.5	Collection lens 2	LA1145	Thorlabs
13 to 14	23.4	—	—	—
14 to 15	15.45	Collection lens 3	LA1805-A	Thorlabs
15 to 16	12.1	—	—	—
16	—	PMT	R12829	Hamamatsu

## Appendix D: Performance of MVPLAPO 2XC

At the preliminary stages of our design, we identified two high-throughput objective lenses: XLFLUOR4X and MVPLAPO 2XC. Our best imaging results were achieved with the XLFLUOR4X. Therefore, this objective was presented in the results of the manuscript. The MVPLAPO 2XC is designed for wide-field illumination in a macrozoom microscope (MVX10, Olympus, Tokyo, Japan), and there have been no reports of its use in a laser scanning microscope. Nonetheless, we analyzed the performance of this objective experimentally using fluorescent microspheres (18859-1, Polysciences, Warrington, USA). The resolution measured with this lens was considerably worse off axis in comparison with the XLFLUOR4X and was therefore not used for LF-TPM (Fig. 12).

## Appendix E: Animal Procedure

All animal studies were approved by the Washington University School of Medicine Animal Studies Committee (protocol #20160217) under guidelines and regulations consistent with the Guide for the Care and Use of Laboratory Animals, Public Health Service Policy on Humane Care and Use of Laboratory Animals, the Animal Welfare Act and Animal Welfare Regulations, and ARRIVE guidelines. To image the cerebral micro-architecture, we removed an approximately 9-mm diameter portion of the mouse skull. Mice were anesthetized with 4.0% isoflurane inhalation in oxygen for induction and 1.5% to 2.0% for surgery. The fur around the incision site was removed with hair removal lotion, and the mouse was placed in a stereotaxic frame to secure the head before surgery. To prevent swelling of the brain, Dexamethasone (2  mg/kg) and Mannitol (20% in 0.9% saline; 200  μL) were administered subcutaneously prior to surgery. Lidocaine was then applied to the surgical region. The skin on the top of the skull was lifted with forceps, cut using sterile scissors, and removed to expose the skull for the craniotomy. Using a dental drill, an ∼9-mm-diameter circle was gently drilled ∼1  mm posterior to Bregma. Drilling was continued until only a thin layer of bone remained. Using thin tip forceps, the skull was removed after applying a drop of saline to the surgery site. Mice were then imaged with the LF-TPM system under 1.5% isoflurane inhalation.
